# Use of research evidence varied in efforts to expand specific pharmacist autonomous prescriptive authority: an evaluation and recommendations to increase research utilization

**DOI:** 10.1186/s12961-021-00789-9

**Published:** 2022-01-03

**Authors:** Akshara Kumar, Amber Bivins Ray, Carrie Blanchard

**Affiliations:** Center for Medication Optimization, University of North Carolina Eshelman School of Pharmacy, Chapel Hill, United States of America

**Keywords:** Pharmacy, Scope of practice, Prescriptive authority, SPIRIT Action Framework, Dissemination, Scope of practice policy

## Abstract

**Background:**

An expanding body of literature shows that pharmacists’ interventions improve health outcomes and are cost-saving. However, diverse state regulations of pharmacists’ scope of practice create a discrepancy between what pharmacists are trained to do and what they legally can do. This study investigated how stakeholders utilized research evidence when developing expanded scope of practice policies in their respective states.

**Methods:**

Using autonomous pharmacist prescriptive authority as a surrogate for general pharmacist scope of practice, a general policy document analysis was performed to understand the scope of practice landscape for pharmacists across the United States. Next, semi-structured interviews with policy-makers and pharmacy advocates were conducted to explore how the identified states in the policy document analysis utilized evidence during the policy-making process. Investigators analysed findings from the transcribed interviews through application of the SPIRIT Action Framework. Resulting codes were summarized across themes, and recommendations to researchers about increasing utilization of research evidence were crafted.

**Results:**

Sixteen states with 27 autonomous pharmacist prescriptive authority policies were identified. Public health need and safety considerations motivated evidence engagement, while key considerations dictating utilization of research included perceptions of research, access to resources and experts, and the successful implementation of similar policy. Research evidence helped to advocate for and set terms for pharmacist prescribing. Barriers to research utilization include stakeholder opposition to pharmacist prescribing, inability to interpret research, and a lack of relevant evidence. Recommendations for researchers include investigating specific metrics to evaluate scope of practice policy, developing relationships between policy-makers and researchers, and leveraging pharmacy practice stakeholders.

**Conclusions:**

Overall, alignment of researcher goals and legislative priorities, coupled with timely communication, may help to increase research evidence engagement in pharmacist scope of practice policy. By addressing these factors regarding research engagement identified in this study, researchers can increase evidence-based scope of practice, which can help to improve patient outcomes, contain costs, and provide pharmacists with the legal infrastructure to practise at the top of their license.

**Supplementary Information:**

The online version contains supplementary material available at 10.1186/s12961-021-00789-9.

## Background

The passage of the Patient Protection and Affordable Care Act in 2010 triggered a massive overhaul of the United States healthcare system by expanding insurance access to millions of people while simultaneously reforming how healthcare services were both delivered and paid for [1, 2]. The influx of the newly insured, coupled with an ageing population, advancing technologies, and more rigorous healthcare standards, exposed entrenched weaknesses in the new system [1–3]. The United States healthcare system was ill-prepared to respond to this transformative new policy, leaving countless Americans without convenient, timely, and quality access to healthcare providers. Nearly 20% of Americans live in areas with a limited number of accessible doctors, especially in rural areas, and the Association of American Medical Colleges projects that by 2030, the demand for new primary care physicians will exceed the supply by over 120,000 [4, 5]. Unmet need for physicians leads to a decline in quality of care, an increase in utilization of high-cost measures, and potential impacts on mortality and morbidity in patients living in these areas [6, 7].

### Expanding health professionals’ scope of practice as one solution

With the looming need for increased healthcare services, a wide range of solutions must be sought to broaden the range of professionals that can safely deliver needed care. One solution that has been effective in times of increased healthcare demand, such as during the worldwide COVID-19 pandemic, has been to expand scope of practice laws for certain healthcare professionals. Scope of practice dictates the services that a healthcare professional can provide to patients, and in the United States, this policy is usually regulated at the state level. During the COVID-19 pandemic, for example, state expansions in scope of practice for healthcare professionals have increased access to testing and treatment, and have allowed states to prepare for future preventive interventions through early licensing to provide the COVID-19 vaccine [8, 9]. Even before this unique public health need, gaps in healthcare delivery had led to the expansion of state scope of practice for a variety of professions, including nurse practitioners, physician assistants, pharmacy technicians, and other healthcare professions [10–16]. Expansions in scope of practice policy allow healthcare practitioners the ability to practice at the top of their field while expanding access to healthcare services.

As evidenced by the pandemic and impact of physician shortages, scope of practice policy falls under the realm of public health policy due to its potential to influence population health and achieve desirable health goals—the definition of public health policy [17, 18]. Though professional practice legislation is influenced by a multitude of political, financial, and economic factors, evidence-based policy-making should also be used in professional scope of practice policy, as it has critical implications in maintaining the safety and quality of public healthcare [18, 19]. Despite its many benefits, effective translation of evidence into policy often is impeded by numerous barriers, which is well-documented in the literature [18, 20–23]. However, these barriers have not been explored in scope of practice policy, specifically in pharmacist scope of practice.

### Pharmacists’ scope of practice

Pharmacists, while historically viewed as simply dispensers of medicine, have been progressively adopting roles as clinical providers due to their expert knowledge in pharmacology and drug treatment [24, 25]. In many states, pharmacists are permitted to perform advanced services, including wellness testing and preventive health measures such as flu testing and immunizations, manage illnesses, perform medication management, administer medications, and provide other transition-of-care services [26, 27]. A growing body of literature shows that pharmacists practicing in these capacities—beyond the traditional dispensing role—lead to improved health outcomes, such as increased access to public health services, improved chronic disease outcomes, and reductions in complications and acute care costs [26, 28–39]. Another example of expanding scope of service includes pharmacist prescribing, which has been shown to be just as effective as physician prescribing in addressing certain chronic disease parameters such as lowering blood pressure or cholesterol [40]. However, despite nationally standardized education and training, their potential to engage in these services and improve healthcare access and outcomes varies based on state scope of practice policy. Studies at both the single-site clinic and state level demonstrate that broader expansion of scope of practice in these areas, such as pharmacist prescribing and disease management, leads to greater access in healthcare and improvements in clinical outcomes [41, 42].

Despite the ample evidence supporting the effectiveness and safety of expanded roles for pharmacists in patient care, inconsistent state-to-state restrictions on pharmacy practice demonstrate a gap between research and effective policy. Policies that are inconsistent with prevailing research hinder pharmacists in some states from delivering care at the top of their license, and have the potential to create impactful discrepancies in healthcare access [43]. One way to reconcile these discrepancies is by improving the utilization and engagement of research by key stakeholders during the policy development and passage process [44]. To our knowledge, no evidence currently exists that details the influence of research evidence during the policy development stage. Understanding how existing research supporting advanced pharmacy services can be leveraged in the development of policy can create an opportunity to better broaden scope of practice through an evidence-based lens.

This study aims to characterize these approaches by investigating the utilization of evidence in formulating scope of practice policy. Specifically, this research explores how policy-makers, including legislators and other members of government entities, and pharmacist advocates interact with evidence when developing autonomous pharmacist prescriptive authority policies. Autonomous prescriptive authority describes the lawful ability for pharmacists to prescribe certain medications based on their own licensing and training requirements, rather than under the license of another prescriber [38, 45]. Allowing pharmacists to prescribe independent of physicians provides a benchmark for other elements of pharmacist scope of practice. Within this realm, the National Alliance of State Pharmacy Associations (NASPA), which advocates for broadening prescriptive authority, identified three areas of existing expanded pharmacist prescriptive authority: (1) contraception access, (2) tobacco cessation, and (3) naloxone access [46–48]. These three category-specific examples are the focus of this study. These policies were specifically chosen as a proxy for identifying states with broader scope of practice, as autonomous prescribing authority is one of the most independent forms of scope of practice, proportionally comparable to independent clinical practice by physicians [19, 49]. Additionally, several organizations, including NASPA and the National Conference of State Legislatures (NCSL), a nonpartisan organization representing the legislative bodies of all 50 states and the District of Columbia, discuss category-specific prescribing as an area of growth and interest among policy-makers and advocates alike [45, 50, 51]. Understanding how research was utilized in and influenced the passage of these existing policies can illuminate effective methods for disseminating evidence for the creation of new evidence-based scope of practice policies.

## Methods

This study sought to examine the connection between established state policies that enhance pharmacy practice and improve public health outcomes through the application of the SPIRIT [Supporting Policy In health with Research: an Interventional Trial] Action Framework [52], described in detail below. Data collection and analysis was conducted from fall 2019 to March 2020. The *first aim* of this study was policy document analysis to identify states with pharmacist autonomous contraception, tobacco cessation, or naloxone prescriptive authority policies passed during the time frame of 2000 to the fall of 2019. The *second aim* was to evaluate the use of research evidence in the development of these policies by applying the SPIRIT Action Framework [52] to the analysis of semi-structured interviews of policy-makers and pharmacist advocates. Finally, the *third aim* was to determine recommendations surrounding conducting research and disseminating evidence in the context of developing pharmacist scope of practice policy based on themes and perspectives derived from the perspectives of interviewees.

### Analytical framework and definitions

**Fig. 1:**
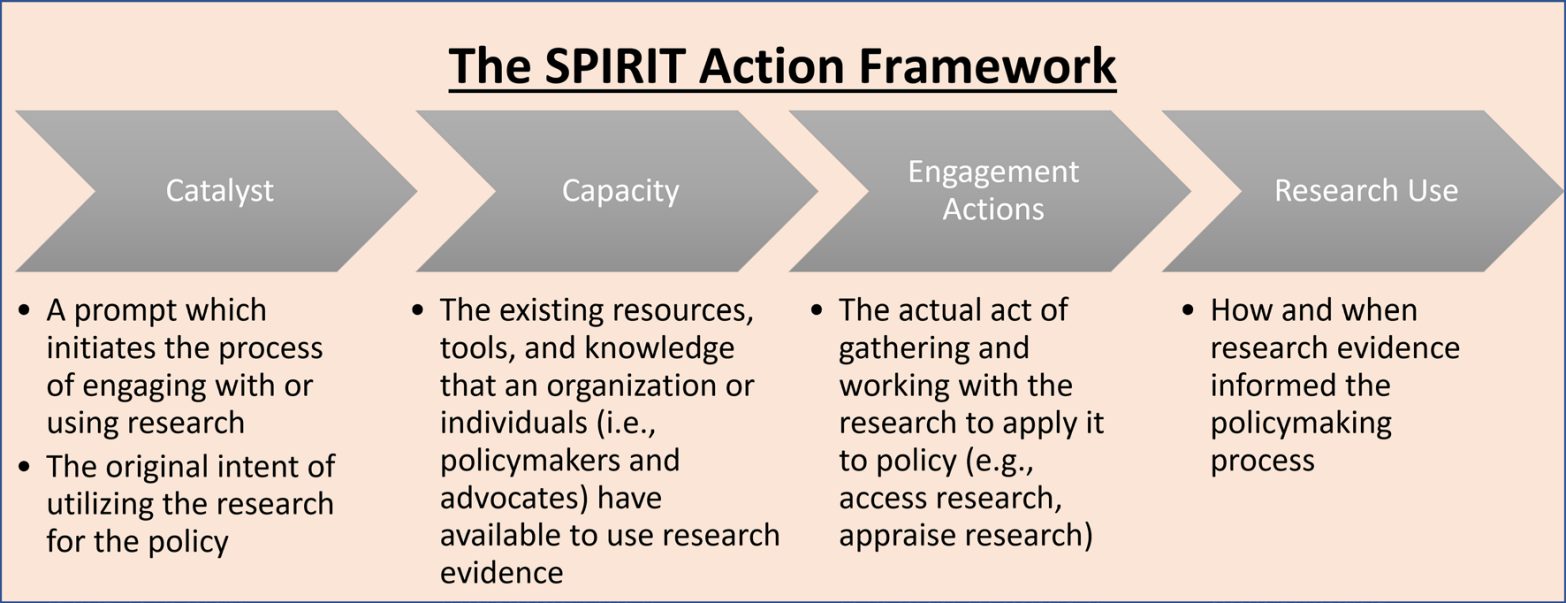
The SPIRIT Action Framework (adapted from the SPIRIT trial [52, 53])

The SPIRIT Action Framework was developed to assess research engagement and influence on health policy and takes into account the role research evidence plays in the context of other factors in policy-making [52, 53]. Operating on the hypothesis that research-informed policies can lead to improved health outcomes, the framework outlines four pillars: *catalyst*, *capacity*, *research engagement*, and *research use* (Fig. 1). A *catalyst* is needed for the use of research, and the response to the catalyst is determined by the capacity of the organization and the individual staff. Where there is sufficient *capacity*, *research engagement* activities must occur that facilitate the *use of this research*. In this study, the SPIRIT Action Framework was used to analyse research evidence utilization in the realm of three categories of autonomous prescriptive authority for pharmacists.

This action framework was designed to analyse the impact of research evidence on policy-makers and organizations that help to draft or develop policies and health programmes [52]. Thus, to determine research evidence engagement and use during the development of prescriptive authority policy, this study considered the perspectives of policy-makers and pharmacist advocates involved in the policy-making process. *Policy-makers* were defined as state legislators who were sponsors of bills, legislation proponents, or agency administrators such as pharmacy board members. *Pharmacist advocates* included members of pharmacy professional associations, such as state chapters of the American Pharmacists Association or the American Society of Health-System Pharmacists.

To fully encompass and assess the use of different types of research, *research evidence* was defined as the analyses of data or concepts found in peer-reviewed papers, technical monographs or books, population trends, grey literature such as internal studies and evaluations, and reports published on government and association websites.

### Data collection

#### Policy document analysis

To identify states that utilize autonomous prescriptive authority, a policy document analysis [54] was conducted to select states which had legislation allowing for autonomous prescribing of contraception, tobacco cessation aids, or naloxone. A combination of resources were utilized, including publications, search engines, and advocacy resources through pharmacy associations, NASPA and the Association of State and Territorial Health Officials (ASTHO) [45, 48, 55–62]. From the states identified in these texts, the prescriptive authority policies were obtained from state legislature websites. To be included, state policies must have been ratified as a law or approved as a rule by 2019 and must have authorized pharmacist independent prescribing of contraception, naloxone, or tobacco cessation. Policy mechanisms included were policies that stated “prescribe”, “furnish”, or “initiate” in their language. Collaborative practice agreements, standing orders, and laws and protocols that only allowed pharmacists to “dispense” were excluded. States that satisfied all the inclusion criteria were identified and were utilized for analysis.

#### Semi-structured interviews: sample recruitment and interview process

Based on the states and policy mechanisms identified through the landscape analysis, investigators sought to interview policy-makers and pharmacy advocates involved in the conception, development, and passage of statewide autonomous pharmacy prescriptive authority in contraception, tobacco cessation, or naloxone. Research ethics approval was obtained by the Institutional Review Board at the University of North Carolina. Through purposive sampling, individuals were recruited through email and phone calls to legislative offices. Interview subjects were selected to create an equal distribution of representation across stakeholder subject types (i.e., legislative body member, pharmacy board or other healthcare agency government member, or pharmacy advocate or association member), prescriptive authority case types (i.e., contraception, tobacco cessation, or naloxone), political affiliation (i.e., Democratic or Republican, which was only applicable for legislative members), and geographic location (i.e., Northeast, South, Midwest, or West [63]). A target sample size of 18 individuals was descriptively identified; within each of the three prescribing categories, at least two states were selected as representative state cases based on interviewer availability. Within each state case, interviews were sought with at least one legislative staff member or policy-maker, one pharmacist advocate or association member involved with the law, and one other individual involved with the development of the prescriptive authority law. For each interview, a semi-structured interview guide (Additional file 1) was developed to explore the use of research utilized during the rule-making process or passage. The interviews were expected to last between 30 and 60 minutes. Specific questions were adapted from items in tools developed from the SPIRIT Action Framework, called the Seeking, Engaging with and Evaluating Research (SEER) and Staff Assessment of enGagement with Evidence (SAGE) tools [64, 65]. After consent was obtained, interviews were conducted and recorded using Zoom Video Communications software, and transcribed verbatim [66]. Though this study aimed for a target sample size of 18, individuals were recruited and interviewed until saturation of responses and perspectives was achieved among various stakeholder groups [67].

#### Coding and data analysis

Using a directed content analysis format, the codebook for analysis of articles and interviews was developed through a theory-derived format based on the elements of the SPIRIT Action Framework [52, 68, 69]. Codes were developed before analysis, based on concepts from the prior SEER and SAGE tools and agreed upon by the research team. Additionally, codes were made to assess recommendations that subjects made for researchers who seek to influence evidence, as well as the types of research and importance of research in relation to the policy discussed. After initial codes were developed, two reviewers analysed the first interview together to reach agreement and common understanding of the codes. Then, a single reviewer analysed the interview transcripts by conducting a first read-through, then a more thorough review and coding. Calibration of the codebook and revision of codes also occurred after continued analysis of the context of the information from the interviews [70]. After all the interviews were analysed by the first reviewer, checks were performed on 20% of the interviews by the third author. The kappa value was based on the two coders coding the presence or absence of 19 codes in each of the transcripts included in the 20% check (i.e., three interviews). The unit of analysis was the transcript; consequently, the kappa value was computed based on the total number of opportunities to agree on the presence or absence of a particular code [69, 71]. Any disagreements that arose after independent coding were discussed and resolved. Coding was confirmed jointly by two researchers on the team.

#### Research team and reflexivity

The research team consisted of a faculty member and pharmacist with significant experience in dissemination and implementation science research and pharmacy policy systems; a health policy fellow and pharmacist with experience in rule-making and public health policy; and a pharmacy student interested in pharmacy advocacy and advancement without prior experience in qualitative analysis. The study was conducted as a part of the pharmacy student’s honours thesis. To ensure an objective and intentional approach, researchers corresponded regularly during data collection and analysis to refine methodology, discuss the application of the action framework, and review and align deduction of salient themes. Researchers also consulted others who were experienced in qualitative thematic analysis to validate methods and utilized the Standards for Reporting Qualitative Research [72] to promote transparency. To standardize the technique in the semi-structured interview, the pharmacy student, who conducted all interviews, was first supervised and guided by the faculty member, then used the interview guide to explore various themes based on respondents’ answers to framework-based questions. These efforts ensured rigour within qualitative analysis and helped to enhance researchers’ reflexivity [73].

To further encourage the balanced influence of researcher and outsider perspectives, codes were also summarized across interviews into an executive summary document and shared with interviewees to validate results as part of a systemized member-checking process [74]. The participants were given two weeks to review the summary, respond to several questions to ensure that their experiences were accurately reflected, and to supplement any additional reflections. Participants’ feedback was cross-referenced with existing codes and was incorporated into deduced themes. Areas where participants disagreed were recognized and reported.

## Results

### Policy document analysis of prescriptive authority policies

**Fig. 2:**
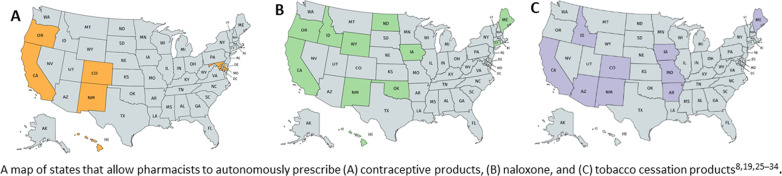
Pharmacist prescriptive authority in the United States. A map of states that allow pharmacists to autonomously prescribe **A** contraceptive products, **B** naloxone, and **C** tobacco cessation products [45–48, 55, 57–62]

The analysis revealed 27 instances of autonomous prescriptive authority in contraception, naloxone, or tobacco cessation spanning 16 different states across the United States (Fig. 2). The mechanism for expansion of scope of practice varied from state to state. Twenty-six of the policies involved authorization via statute, which referred to either the creation of a new law or amendment of an existing act authorizing pharmacy practice in the respective state. Additionally, 17 of those statutes were implemented via a pharmacy board rule or statewide protocol; only one state exclusively used a rule instead of a statute to authorize prescribing.

### Characteristics of participants in semi-structured interviews

In total, 14 individuals participated in semi-structured interviews regarding the use of research evidence in pharmacist prescriptive authority. Interviews lasted between 30 and 60 minutes on average. The distribution of subjects by interviewee and prescriptive authority type, as well as the characteristics of state cases, is outlined in Table 1.

**Table 1:** Interviewed participants’ characteristics

Interviewee type	Description	Count (*N* = 14)
Association members	Administrators of pharmacist professional associations such as state chapters of the American Pharmacists Association or American Society of Health-System Pharmacists	5
Agency members	Administrators of government organizations, such as the department of health, or pharmacy boards	5
Legislators	Lawmakers who sponsored the prescriptive authority statute in their legislature	4
Democrat		2
Republican		2
By prescriptive authority type		
Contraception		5
Naloxone		5
Tobacco cessation		4

### Analysis of interviews through application of the SPIRIT Action Framework

Overall, the interviews provided insight into how legislators, association members, and agency members engaged with research evidence to develop and advocate for prescriptive authority policy. Types of evidence that were used for policy-making included statistical data and population trends, published peer-reviewed articles and meta-analyses from prominent pharmacy journals, and clinical data and guidelines. The following sections present the findings framed within the various pillars of the SPIRIT Action Framework. Analysis of interview responses and inter-rater reliability was verified with a calculated kappa coefficient of 0.78.

#### Catalyst: public health need and safety concerns motivate utilization of research

The SPIRIT Action Framework [52] defined the first component for evidence utilization, “catalyst,” as a prompt that occurs to initiate the process of engaging with or using the research to influence the prescriptive authority policy. The major themes in this category from participant interviews are summarized in Table 2. Overwhelmingly, most respondents commented that public health need was the primary factor in driving research utilization. Interviewees cited data regarding access to care and public health concerns (e.g., high rates of unintended pregnancies, smoking rates, and opioid-related deaths in their states).

**Table 2:** Summary of themes describing the “catalyst” for research use in prescriptive authority

Framework sub-concept	Theme in the context of prescriptive authority	Quote
Inform understanding of an issue	Safety and efficacy of pharmacist prescribing	“So we had to be able to marshal our facts and have all the information available and be able to convince people that the data were overwhelmingly clear that this was a safe thing to do.” “It was definitely part of, kind of, again, that foundational research body… in terms of looking at it basically was the basis for a while, he said there is advocacy, you know, it is a promising practice to use pharmacist to conduct this work.”
Establish a need for a policy	Public health need	“The need for naloxone was quite evident in information and resources that we use and found where [our state] was second from highest for opioid overdose death rates… it was clearly a public health concern and an area where pharmacists should be involved.”
	Physician shortage	“…the data that was used was just pointing to the primary care shortages and being able to point to that as a need for expanded care.”
Influence the text of a policy	Successful precedents were followed	“There were others who actually reviewed that data of other similar programmes and brought information from, anecdotal information, from other states to that failed, and that helped to inform us in making this programme as successful as it has been.” “[Our state] had a long history of allowing pharmacists to do—to give immunizations, for example, we’ve been doing that for a really long time for people 10 and above, and then… we pass legislation that saying in the event of a public health emergency they could give them to kids as young as three. So we feel pretty strongly that pharmacists… in general has pretty wide scope of practice laws.”

Interviewees also cited research establishing the safety of having pharmacists providing prescriptive services for these medications as another catalyst. Designating prescribing to professionals other than physicians often required extensive advocacy and discussion regarding the capability and expertise of these professions. As one legislator said regarding contraceptive prescriptive authority:

[W]henever you make a major change like this, especially when it’s something around reproductive health…you have to really have your ducks in a row. So without the right, the solid evidence to support it, we knew we weren’t going to get anywhere. So we had to be able to marshal our facts and have all the information available and be able to convince people that the data were overwhelmingly clear that this was a safe thing to do.

To this end, policy-makers often looked to other states’ data regarding evidence of successful prescriptive authority policy development to inform new policies in their state.

#### Capacity: components that supported research evidence use

The SPIRIT Action Framework [52] identified the existing resources, tools, and knowledge that an organization or individual has to utilize research evidence as the “capacity” for research engagement. Table 3 presents the themes for capacity: individual and organizational value of research, skills and knowledge for acquiring and applying research, the availability of resources to access research, and the staff and person power to utilize research.

**Table 3:** Summary and quote examples of themes enhancing “capacity” for research use

Framework sub-concept	Theme in the context of prescriptive authority	Quote
Value of research	Value of research is present among individuals and organizations whether research evidence was used or not	“It’s pretty important to have it…[and] that’s pretty clear for us at that whole board. One of the things we wanted to do was to make sure that every decision that we made was as evidence-based as it could be.”
Skills and knowledge	Healthcare providers’ familiarity with interpreting research enhanced capacity	“Having two physicians, one in the House and one in the Senate, to, you know, help write the policy, and then also you are the primary advocates for it in the legislature was very helpful because it brought subject matter expertise… As scientists and physicians, we are trained to solve the problem using data, research and trial and error.”
Resources to access research	Emphasis on recognizing publicly accessible research resources as opposed to private sources for research evidence	“[H]e starts his PubMed searches and things like that to find out who’s got published data on any given topic… he’s on the editorial board for APhA [American Pharmacists Association]’s journal.”
Staff and manpower	Staff and students enhance potential for using research	“[A]s far as actually doing the research or getting the research, a lot of that was done by board staff and if we had P4 pharmacy students on rotation at the time…the board itself relied heavily on board staff to do a lot of that research and present the findings.”

Many individuals interviewed cited a strong regard for research from both their personal perspective as well as on behalf of their policy-making organization. Despite the recognized importance of research in policy-making, interviewees acknowledged that research sometimes did not play a role in the policy-making process, which was not aligned with their own value of research. Others discussed how research evidence is important for advancing pharmacy practice and developing other prescriptive authority policies for their state.

In addition to value, interviewees cited that having a healthcare background or training helped to increase the capacity to use research. Healthcare professionals, such as those in professional associations, or legislators with experience in healthcare enhanced research evidence use because of their ability to analyse and apply data.

Participants also cited how different resources helped to increase evidence use capacity. When describing evidence that was available to them, publicly available research (e.g., news articles, professional organization materials, and open-access journals) was identified more frequently than restricted-access sources (e.g., internal evidence from a governmental department, standard subscription journals). Additionally, capacity to use these resources was closely tied to the availability of staff members and their ability to engage and apply research evidence. Specifically, a few participants discussed having other staffers and/or fourth-year pharmacy students as key to their capacity to utilize research.

#### Research engagement actions: using resources and experts to access and apply research

Highlighted by the SPIRIT Action Framework as a bridge between “capacity” and the outcome of research application, the actual collection of evidence and interaction with research was defined by the “research engagement actions”. As shown in Table 4, four components were considered based on the action framework.

**Table 4:** Summary and quote examples of themes regarding “research engagement actions”

Framework sub-concept	Theme in the context of prescriptive authority	Quote
Access research	Prioritized easily accessible research	“…most of them are peer-reviewed journals. Some of them are not but they still published whatever somebody else found out some place else or, you know, the American Pharmacists Association has research and papers presented at their annual meetings” “I think with every policy we scan the nation to see who was doing what and… I would say that we did look out to see who else was prescribing naloxone.” “…the demographics of [our state], the department of health publishes the demographics on the overdose death rate, what the products are, and that sort of thing, so we used their research.” “…there was also just—there were some articles, I don’t know that I read the study, but they’re articles about how…there were more people using contraception [in another state] after their legislation had been enacted and implemented.”
Appraise research	Evaluated *relevance* and *significance* of research evidence	“We were very deliberate in talking through… primary differences between the California model and New Mexico’s model and our model… California only allows prescriptive authority for the nicotine replacement therapy products. They’ve got all five, they include the over-the-counter products as well as the prescription inhaler nasal spray. But there was quite a bit of back and forth, especially with the physicians on the group. Are there are their concerns and even though black box warnings, the black box warning that removed…. And that was something where we have these very engaged discussions, and it kind of came out that, you know, these products when use is recommended are FDA [Food and Drug Administration]-approved and they’re generally safe. And so we wanted to include all seven.”
Interact with researchers	Interacted with research-knowledgeable individuals	“…about 30 stakeholder groups, local health departments, health insurers, pharmacists, pharmacies…” “We did have the expert [from the department of health] with us, and she utilizes those statistics to, you know, kind of back up the evidence that we had cited.”
	Individual and organizational opinion were valued equally	“The nurses association, the pharmacist, you know, so we had a lot of positive testimony… their organization supported this and they support it because… they thought it was the right thing to do based on the scientific evidence.”
Generate new research	Not often utilized to engage with research evidence	“So we did a statewide survey that basically looked at…if this legislation was passed, you know, essentially what, what direction [will] we go into the protocols that were selected… through the statewide survey…. So we had physicians, physician assistants…respond to this… and then we did have non-clinicians answer so, you know, essentially health plan or payer staff, public health staff, and that sort of thing and kind of get a broad cross section.”

##### Access research

Convenient, familiar sources were used by association members and policy-makers. The most commonly referenced sources were ones that could be accessed through search engines and that respondents recognized through their professional experiences and connections. Databases referenced included PubMed, Google Scholar, and the Cochrane Library. Academic and specialized resources also were used for policy-making, such as the Surgeon General’s guidelines for tobacco cessation interventions or the American College of Obstetrics and Gynecology’s (ACOG) contraception guidelines. In addition, some individuals also reported having access to public health data from their state’s department of health or unpublished data from other states that had implemented pharmacist prescriptive authority.

##### Appraise research

The relevance and significance of the research was important to its use in policy-making. Some respondents reported carefully evaluating research and public health data from other states and countries to ensure applicability and impact to their own state’s policy. Additionally, if the research advocated for an actionable direction, or if interviewees found the findings “compelling”, as some interviewees noted, it helped to motivate policy development in that direction. These concepts of relevance, the potential to see similar impact in their own states, and clear recommendations illuminated by the research dictated engagement with the evidence.

##### Interact with researchers

The SPIRIT Action Framework emphasized “interactions with researchers” as a component of its “research engagement actions;” however, participants in this study reported engaging with research-knowledgeable experts and stakeholders who helped to present and summarize the available research for policy-makers. These brokers of evidence knowledge were found in special interest organizations, such as Planned Parenthood or harm reduction coalitions, or with professional organizations such as physician or pharmacist groups. Individual practitioner testimony was also utilized for their key insights into research and their ability to connect the research to actionable policy-making for legislators.

##### Generate new research

Generation of new research for the purposes of policy-making is another component of the “research engagement actions” pillar. When interviewees were asked specifically about this, only those from one state described conducting research for the purpose of influencing their prescriptive authority policy.

#### Research use: advocate for safety and influence key components of policy

Finally, the SPIRIT Action Framework “research use” pillar described how the research informed the policy. This pillar was composed of two main components—how research was used (i.e., conceptual, instrumental, tactical, or imposed fashions) and when it was used (i.e., time in the policy-making process). The themes are summarized in Table 5.

**Table 5:** Summary of themes describing “research use” in pharmacist prescriptive authority

Framework sub-concept		Theme in the context of prescriptive authority	Quote
Purpose of research use	Conceptual: To provide new ideas, understanding, or concepts	Used to understand different considerations for prescriptive authority policy	“…that our biggest concern, of course, is, you know, around *safety*… is this a practice that is going to be *beneficial* for women’s health and not hurt people. And the data is pretty clear with regards to that experience.” “…one of the things, you know, that research shows us was that there are—one of the barriers for pharmacists actually being able to provide this service is *the barrier of getting compensated for the service, reimbursement*.”
	Instrumental: To directly influence the content or wording of a policy	Used to inform components of the policy itself	“Our adult immunization rates in [our state] were horrible; I’d say they’re probably the best of the best in the nation now, because of the programme… And so we would basically *take that same protocol that we had for immunizations and we would go in and insert in and put in naloxone therapy*.” “We did kind of look at that model as far as how that may work as far as *the education, the training* that we want to get pharmacists involved in.”
	Tactical: To justify or lend weight to pre-existing ideas	Used to advocate to healthcare providers and legislators	“When we were meeting with some of the folks, in particular, [our state’s] medical association, we did have publications, you know, showing how pharmacists in different settings have helped patients with tobacco cessation.”
	Imposed: To meet an organizational requirement	Not used very often to motivate research use	“We had to have documentation from department of health about what the problem was that was research from them, in terms of state demographics of overdose death rates and that it was being caused by prescription drugs.”
Timing of research use	Agenda-setting	Helped to assess the need and feasibility to prioritize policy	“But we did find in [our state], that the bandwidth of the pharmacies, even to do that brief intervention, because it's not just conducting it, it’s privacy concerns… it’s documentation. It’s the whole process of logistics of actually doing the referral. And so it ended up kind of getting tabled.” “…needed desperately to get out of being the worst in the country in overdose deaths…”
	Policy development	Helped to guide policy direction and wording	“[the staff member who looked at research evidence for policy] did a lot of the research and a lot of the wordsmithing outside of the meetings, and he would bring us the final documents that we would review…”
	Policy implementation, monitoring, and evaluation	Helped to look at several metrics to understand impact of policy	“And after we had done this, we looked at *how many had been prescribed after hours on holidays or on weekends* when prescribers are typically closed… But what we found was what we thought would be true. And we found that was that the pharmacists are the most accessible healthcare professional.” “So the protocol for prescriptive authority was not deemed effective. It did not increase item movement. It did not decrease opioid death rates, opioid overdose death rates in New Mexico. So it was not statistically or clinically significant…. We did see, I don’t want to say, a statistically significant number in naloxone is given out at the pharmacy level, but an increase.”

##### How was research used in prescriptive authority?

Broadly, research evidence and public health data were used to understand the considerations necessary for allowing pharmacists to prescribe these products. Specifically, participants described using research evidence to conceptualize the safety and efficacy of pharmacist prescribing, barriers to implementation from prior prescriptive authority policies, and the public health issue at hand.

Research evidence and data from successful prior prescriptive authority policies also helped to dictate the specific components included in the participants’ states’ policies. Pharmacy advocates and legislators alike discussed how successful pharmacist immunization policies helped to dictate the formatting of subsequent prescriptive authority policies. Studies also supported the use of other aspects of the policies, such as incorporating reimbursement methods and certain training requirements. Finally, participants used research evidence to identify specific products pharmacists could prescribe and other requirements to provide complete care. In one state, studies highlighting the effectiveness of the Quitline, the national telephone-based tobacco cessation service, supported its incorporation into prescribing authority policy, where pharmacists also had to refer patients to this service.

Another use for research was advocacy for participants’ policies allowing pharmacists to prescribe. Interviewees and news articles reflected the use of evidence when defending the legislation against opposing stakeholders. In one case, a pharmacy advocate even employed research evidence engagement as a tool to argue for the safety of pharmacists compared to the risks associated with these public health concerns:

We basically focused on, is the risk of smoking higher than the risk of having a pharmacist prescribe smoking cessation products…we challenged them to … show us something…in the literature that shows us that having a pharmacist assist with tobacco cessation went horribly wrong…and then we will put our data up…against your data, and that…kind of helped us defend our position.

Clinical data and research evidence were also used to advocate for the feasibility of autonomous pharmacist prescribing. For example, legislators used a study called the Direct Access study to support the ease of selecting contraception based on patient factors [40]. This, along with clinical guidance from ACOG, helped support having pharmacists prescribe contraception rather than doctors. Similarly, California and Colorado turned to research-driven tobacco cessation pilot programmes to demonstrate the successful implementation of tobacco cessation interventions and product selection by pharmacists on a smaller scale [41]. According to interviewees, these forms of evidence engagement helped to successfully advocate for prescriptive authority.

A few respondents specifically discussed how research evidence engagement was required due to a state mandate or a funding requirement. However, this was not common, and research was primarily used for the substance it provided to policy-making.

##### When was research used in prescriptive authority?

Evidence and research were used throughout the policy-making process. Research evidence helped to prioritize policy and set the legislative agenda by comparing the need for the prescriptive authority policy versus its feasibility of implementation. Additionally, research evidence was used throughout the policy development process, from helping to direct the details and wording of the policies to dictating the processes whereby pharmacists prescribed and documented their services. Finally, respondents described collecting evidence and analysing metrics of uptake of pharmacist prescribing and medication access after policies were passed, demonstrating the use of research evidence to monitor the implementation and impact of prescriptive authority policies.

#### Barriers to utilizing research evidence in prescriptive authority policy

Though participants spoke to most of the components of the SPIRIT Action Framework and discussed their engagement with research evidence, it was evident that research evidence did not always play a role in policy-making. When participants were asked to rate the importance of research in their prescriptive authority policy on a scale of 0 (low importance) to 5 (high importance), the average rating was 3.2 (standard deviation of 1.5). Participants were asked to identify barriers to engaging with research evidence. These barriers were categorized into two groups, described in Table 6.

**Table 6:** Barriers for research evidence engagement in pharmacist prescriptive authority

Sub-concept	Theme in the context of prescriptive authority	Quote
Individual barriers: innate barriers that prevent the individual from utilizing research evidence		
Lack of skill set	Lack of ability to translate research into actionable policy	“I think the big, I guess, the barriers if I had to call them out or kind of get at how the model is going to be translated, are going to have different workflow challenges, they’re going to again have different capacity challenges. I think the big part wasn’t so much developing the protocol as the work that’s happening, literally right now it’s how does that protocol gets translated.” “…the barrier to using real research is that it is difficult for the average person to understand.”
Lack of time	Inability to designate enough time	“…how much lead time you give yourself with regard to crafting legislation, you know, that I can think of a couple of other bills that I've worked on for over a period of time where you do kind of have a little bit of space to be able to use the expertise of researchers. But in this instance, I did not have [that time].”
Contextual barriers: factors outside of the individual’s or organization’s influence that prevent utilization of research evidence		
Research was not there	Applicable research was not available at the time to guide policy	“It’s still relatively new. So we were one of the first, you know, handful of states to actually implement the policy…sometimes policy get ahead of where research is, yeah, and so, you know, that potential obstacle.” “…sometimes there’s not great data. You know, the studies are small so they have their study limitations, all the same ones we always hear, studies small, you know, not enough numbers in power, you know …you know, it wasn’t…it wasn’t the double-blinded double.”
External stakeholder opposition	External opposition led to compromised practice	“So turf battles with medicine is pretty popular opposition that we face for a lot of these … So that’s probably, you know, that sticks out in my mind sort of the first—the first and most prominent of the hurdles that we face because we face it every time we—every time we try to do this.”
Lack of value	Research evidence was not as highly valued as other considerations	“Some of them like research but most of them like anecdotal stories, so if you can get somebody to come in and say ‘I overdosed on this and I wish someone gave me naloxone and revived me’, that’s a good story. Works better than hard research sometimes.”
Catalyst did not exist	Pharmacists were the “logical” solution	“It was looked at as a pretty common-sense approach to allow pharmacists to prescribe, you know, what is, you know, pretty universally seen as a safe medication… I don’t know if there would really be able to point to much that I would say that this is what really drove it home or anything else.”
	The urgent need for the policy outweighed research use	“There wasn’t a lot to research at the time, and the value of developing the policy importance is still…important that we do it. That’s not fair, but it’s what I feel.” “…prescriptive authority for oral contraceptives or prescriptive authority for smoking cessation products and that, I mean, you know, we can we can make some… arguments for and against that…but when you look at naloxone specifically, I mean, the goal here is to save people’s lives, you know, in that respect, and take care of an epidemic that was…you know, we’re right in the middle of, so it just was not a controversial item.”

##### Individual barriers

Individual barriers describe the innate obstacles a policy-maker or advocate may have faced in utilizing and translating evidence into actionable policy. A lack of knowledge or skills to apply research evidence to policy prevented some participants from using research evidence to advocate for legislative change and expanding pharmacist practice. Additionally, one participant argued that for some policies it is hard for legislators to allocate enough time to find and interpret research evidence for policy.

##### Contextual barriers

###### Lack of high-quality, applicable research

One barrier that prevented research engagement outside of individual skills was the lack of research. Many participants discussed how prescriptive authority for pharmacists was a novel concept, and before policies like this were passed, there was no research testing or proof of concept study. Several commented that their states were among the first to allow for pharmacist prescribing, limiting the amount of data that they had available to use.

Even when evidence was present, the lack of relevant data prevented the use of research evidence in prescriptive authority. Small sample sizes for studies, limited double-blinded trials, and research that did not discuss the policy at hand (e.g., utilizing evidence of successful contraceptive prescriptive authority for tobacco cessation policy) were cited as reasons for inapplicable data.

###### Other influences on scope of practice policy had a more impactful role

For some participants, the ultimate barrier to utilizing research was that it was a secondary goal compared to other influences on prescriptive authority policy. The political opposition of scope of practice policy, for example, prevented the optimal use of research evidence in prescriptive authority policy. Subjects often noted that while they themselves knew of the evidence supporting pharmacist prescriptive authority, opposing stakeholders were against broadening pharmacist scope of practice. This caused policy-makers to compromise on policy even when it did not reflect the supporting evidence of what pharmacists could do. Examples of this included limiting pharmacists to prescribe only over-the-counter medications instead of effective prescription medications or limiting the age of patients that could be prescribed contraception.

Like political opposition, research evidence was sometimes not as valued by other stakeholders and thus not always an effective advocacy tool. Some legislators commented that anecdotal stories (i.e., personal testimonials from patients or clinicians) played a larger role in influencing policy, since legislators could better empathize and relate to it. Some interviewees attributed this to lawmakers’ backgrounds being primarily in fields other than science, preventing them from evaluating the significance of the data over financial considerations or anecdotes from their constituents.

###### Research may have just been unnecessary for this public health policy

At its core, pharmacist prescriptive authority in the areas of contraception, tobacco cessation, and naloxone were often viewed as solutions to a public health need, with the added benefit of advancing pharmacist practice. For this reason, some interviewees noted that in these situations, policy development could not wait for available research; the urgency of the public health need to mitigate the problem took precedence over research evidence. For some individuals, especially association and pharmacy board administrators, pharmacists “just make sense” when it came to solving a public health concern; they used the pharmacists’ expertise and accessibility to logically justify the policy. Regardless, limited time and urgent need for developing a policy solution was a barrier for research utilization across all prescriptive authority categories.

#### Recommendations from participants to increase research evidence engagement

Based on their experience, interviewees recommended ways to increase research evidence engagement (Table 7).

**Table 7:** Respondent recommendations for research evidence engagement in pharmacist prescriptive authority

Recommendation	Quote
Research should be clearly relevant to policy-makers’ goals	“I think that it’s sort of keeping up with where different policy trends are or major issue areas are and then reaching out to people who—to legislators to make them aware of their research would be helpful.”
Maintain connections with policy-makers	“You can’t just, like, show up when you need something, so to create a network and a relationship with, like, influencers prior to and even…creating that relationship. So that to me is the most important thing.”
Tailor research dissemination to audience	“…about sending, you know, about press releases that talk about the practical implications of this research…” “look at research and site numbers, especially if you have nice visual like graphs or tables, that always helps make a stronger case…. Remember that their audience is…probably is not going to be research-oriented and not going to be an academic. So while of course you still want a really robust study design, it really comes out on the back end to think about how that evidence is portrayed for potential lay-people.”
Leverage stakeholders	“And I think that in every state there is a college of pharmacy… you know, state college of pharmacy is indeed a great resource for legislators who are interested in such things.”
Continue to build evidence base	“Publish often, publish more… even if the research showed, hey, this didn’t work. That would be useful too because there just really was…you know, there’s not always a lot of data.”

##### Research evidence should be clearly relevant to policy-makers’ goals

Lawmakers and association and agency members commented extensively on the need to measure specific outcomes and sustainable payment mechanisms to produce research evidence that is relevant for policy-makers. Many interviewees considered real-life scenarios and barriers that patients may face, prescribing rates, and the practical implications surrounding pharmacist prescribing practice. Some even suggested creating pilot programmes with constituents to test a potential policy and its impact prior to passage. Quotes regarding some of the key metrics that participants were interested in are outlined in Table 8.

**Table 8:** Recommended metrics for further research in evaluating prescriptive authority efficacy

Metrics	Example quotes
Demographic data on impacted populations	“So I definitely think interviewing the impacted populations and, you know, *getting as much data [as] you can on the population that you're servicing* and your service area community.” “But I think at this point it is a lot of state research, there are a number of states that are doing naloxone so I suggest they see *what other states are doing now and what their demographics are*.”
Usage of pharmacist prescribing practices	“…then if it is passed, did it have its *intended effects as measured by outcome studies and in that process demand*, you know, what are the key outcome measurements that we need to track over time.” “And what is *the referral rate*…physicians are very worried about fragmented care.” “…one of the common questions… is like, well, if that state did it, how did it turn out? And so we need more post-data collection…because so many times we're like, was it successful? *How many did we prescribe, and how did it change things*?”
Cost and payment	“proving of… their ability to *potentially save the healthcare system dollars* is something that I think is instrumental, you know” “I think for pharmacist uptake *research around payment*…”
Workflow evaluations	“Are there any *safety considerations* or reports to the board about *any issues related to pharmacists prescribing*?”

Additionally, interviewees found monitoring the practical implications of these policies important for future policy development. Numerous interviewees discussed how monitoring and evaluating policy impact post-implementation, for their own or other states’ prescriptive authority policies, was critical. One participant emphasized the importance of demonstrating the efficacy of these policies for future broadening of scope:

I think research is critical in the reality of moving beyond naloxone, you know, I think you’ll probably talk to other states where, you know, they’ve expanded their prescriptive authority beyond naloxone, and I think that’s really where, you know, pharmacists have a lot of opportunity…providing that research behind in some of those states where it has been effective and have been implemented, then that could really build the ladder for other states to take that on as well.

##### Maintain connections before and after researchers influence policy

Multiple interviewees also recommended that researchers maintain strong relationships with policy-makers and advocates throughout the research pipeline—from conceptualization to dissemination. Increasing awareness among legislators for potentially policy-relevant studies can overcome legislative time restrictions and support evidence use in prescriptive authority policy. Other pharmacy advocates also recommended that researchers seek opportunities to converse with policy-makers and pharmacy policy influencers outside of legislative sessions and other policy-making meetings. Overall, interviewees noted that because of the barriers to actively accessing and being aware of evidence, researchers should maintain connections and engage policy-makers in research development and new findings as they appear, not simply when lawmakers are in session.

##### Tailor research dissemination towards audience

Multiple interviewees commented that, when disseminating evidence, researchers needed to be cognizant of their audience. Interviewees discussed how to better formulate evidence to reach legislators who are either pressed for time or who do not have the same capacity to interpret and apply literature as other researchers. One association member discussed utilizing graphs and charts for portraying key takeaways to make the data more accessible to policy-makers. Interviewees noted that even when studies have a robust design and support policy goals, researchers should also convey these findings in non-peer-reviewed resources that can be easily accessed and interpreted by those who are not research-oriented.

##### Leverage stakeholders to conduct and disseminate research evidence

Participants also discussed the value of collaboration when advocating for evidence-based expansion of scope of practice. Utilizing national organizations and policy advocate groups to relay research to lawmakers was strongly encouraged by stakeholders. Organizations such as NCSL provided resources to legislators at the state level, creating an identifiable channel for making research evidence more accessible. Additionally, interviewees also discussed the use of pharmacists in academia and practice to help conduct research and expand the available data on pharmacists’ impact in scope of practice. Schools of pharmacy were cited by some as a potential resource for creating evidence and monitoring the impact of new policies. Lastly, one participant described using community pharmacists and residents to monitor implementation of scope of practice, as they can document the uptake of prescribing and barriers to providing services.

##### Building the evidence base on prescriptive authority and expanded pharmacist practice

Regardless of the findings of a study, some participants identified that all data regarding development of scope of practice policy was valuable. Thus, interviewees recommended that researchers amass as much research evidence as possible on broadening scope of practice, as it could help future decisions regarding pharmacists’ scope of practice. One participant even stated that researchers should work to make all evidence, regardless of the rigour or formal design of the study, available to legislators and advocates to inform decision-making.

## Discussion

This study investigated factors that influence research evidence utilization among policy-makers and pharmacist advocates during the creation and passage of pharmacist prescriptive authority policies. Laws were found to be the primary mechanism for policy-making surrounding pharmacist prescriptive authority, indicating that legislative policy-makers are key arbiters of pharmacist scope of practice. Key motivating factors for research evidence engagement included addressing a public health need, evaluating the safety and efficacy of expanding pharmacist practice, and examining the successes and challenges of similar policy. Additionally, the capacity to utilize research was influenced by the value of research, familiarity with analysing and applying evidence, and access to resources and staff. Respondents discussed how the actual act of engaging with research involved easily accessible sources and interactions with research-knowledgeable individuals. Research evidence was appraised for policy use by assessing its relevance and significance. While research evidence was used throughout the policy-making process, from agenda-setting to post-passage policy monitoring, respondents also utilized research evidence to comprehend the issue at hand, to dictate policy wording, and to advocate for the policy to others. Respondents reflected on a lack of time and skill set that limited their capacity to utilize research, while also addressing situational obstacles such as research availability, value of evidence, and the urgent need for a policy. Nevertheless, respondents were able to contribute several recommendations to researchers who seek to utilize their work to influence policy, encouraging its importance and use in evidence-based scope of practice.

To our knowledge, this is the first study that assesses the influence of research evidence on the development of pharmacist scope of practice policy. Despite this lack of literature on pharmacist practice policy, literature surrounding nurse scope of practice and advocacy has been a key legislative issue for nurses for many years. Nursing organizations have used research findings supporting expanding scope of practice by creating key advocacy materials to help promote their profession [75, 76] and garnering support from other medical institutions such as the Institute of Medicine (now the National Academy of Medicine) [77]. While this is not yet true for pharmacists, this study identifies key factors that can foster engagement with research evidence in encouraging broader scope of practice. Like nurses’ scope of practice, advocacy methods that capitalize on the research evidence can potentially help in moving forward not only pharmacist practice and patient care, but also the implementation of evidence-based health policy and practice.

This study has some limitations that must be considered when examining the implications of its findings. While our study had a small sample of interviewees, there was equal representation across different stakeholder types, political parties, and prescriptive authority types, increasing the study’s generalizability. Additionally, another potential limitation is the subjectivity inherent in the qualitative analysis study design and findings. To mitigate the biases of the authors, synthesized member-checking was conducted and a reflection on the reflexivity of the study design was addressed. Statistical verification of inter-rater reliability through determination of the kappa coefficient, in addition to member checking, helped to establish both the trustworthiness and the external validity and transferability of this thematic analysis. Overall, despite the presence of limitations, this study optimized a purposeful representation of policy-makers and pharmacy advocates to create a generalizable characterization of research engagement use in prescriptive authority policy-making.

**Fig. 3:**
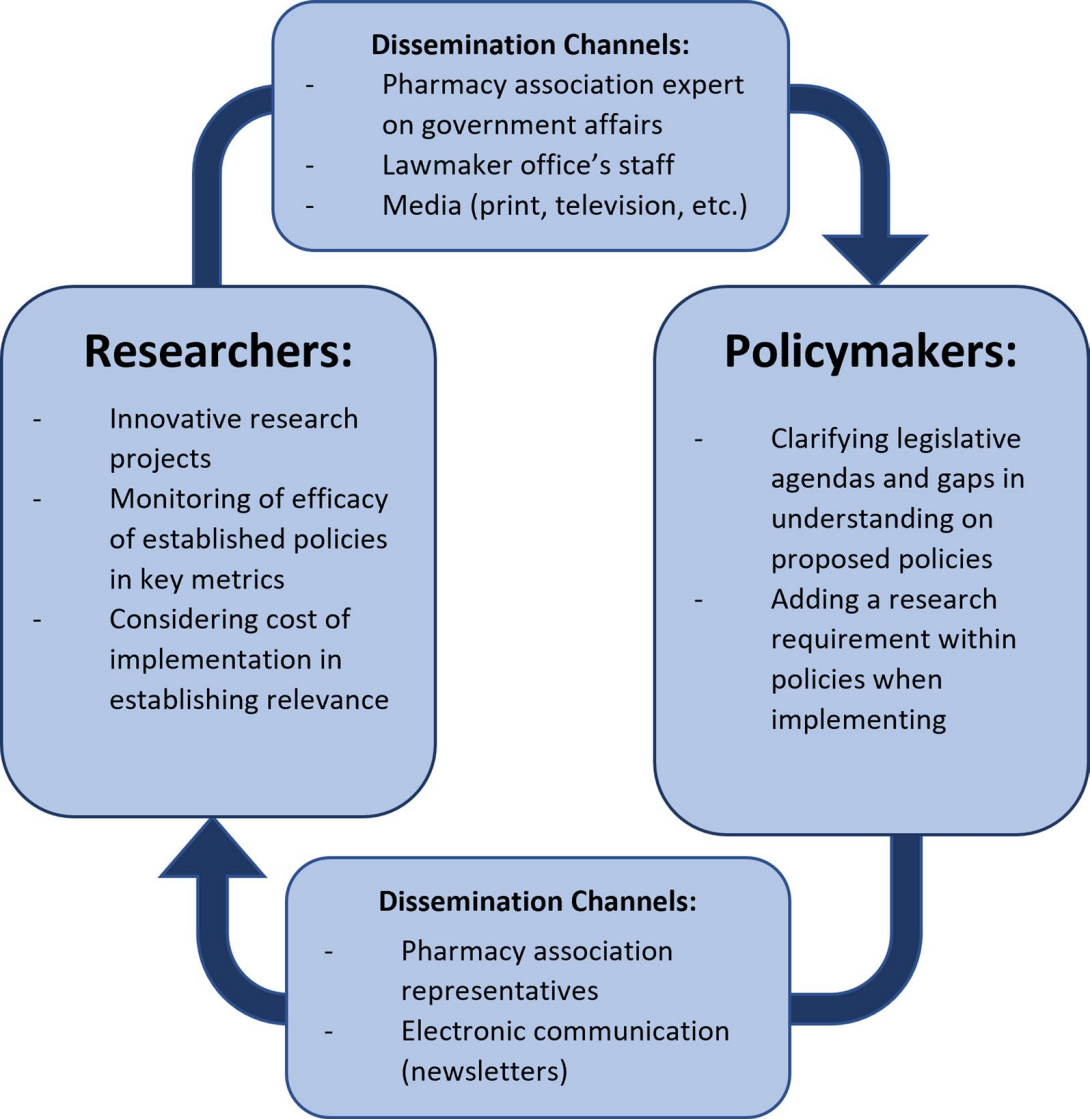
A potential model for the researcher–policy-maker relationship

This study highlights that there is room to improve the dissemination and implementation of research to expand scope of practice for pharmacists. As identified by respondents, a multitude of other factors, from constituent anecdotes to political opposition, compete with the consideration of evidence in policy-making. To better increase the significance of their work, researchers investigating the impact of advanced pharmacy practice can use a number of initiatives to enhance policy-maker engagement with their studies. First, researchers may need to better understand the policy-making process and the considerations of expanding scope of practice, to find ways in which they can contextualize their studies with other policy influences. As respondents noted, not only do researchers need to form relationships with policy-makers early in the policy-making process, but these relationships also need to be well established and reciprocal. A potential model for the policy-maker–researcher relationship is depicted in Fig. 3. By enhancing these relationships, researchers can easily assess research needs or present research that is relevant to upcoming policy. Conversely, policy-makers can utilize researchers to analyse the impact that their policies are having on their constituents, which can serve as evidence of effective policy solutions for other policy-makers in other communities. Both researchers and policy-makers can optimize the use of resources and stakeholders who understand the government process to help strengthen channels for communication. This proposed dynamic model supplements existing literature emphasizing the integration of researchers in the policy-making process, but, using the context of pharmacy policy, expands upon the need for improved communication mechanisms between policy-makers and researchers [78–80]. Overall, the policy-maker–researcher relationship, when established in advance of a policy need, can chronicle the successes and challenges of policy-making and help to enhance evidence-based scope of practice expansion in a way that demonstratively benefits patients.

Researchers seeking to influence scope of practice policy must also consider the method of dissemination. As outlined by respondents, policy-makers without a medical or science background have difficulty accessing, interpreting, and prioritizing research evidence in policy-making. It has been shown that social media has enhanced the dissemination of knowledge and has continued to be explored as a tool for disseminating research [81–83]. Researchers can take advantage of social media, traditional media, and other easily accessible sources that legislators may be more likely to engage. Additionally, researchers can consider portraying their results in novel ways to enhance readership and understanding of the implications of their work. Creating reviews of the literature in a policy paper format, for example, may help to frame research in a format that lawmakers are more familiar with, comparing the advantages and pitfalls of certain models of practice. Blog posts, visual abstracts, and other resources that help to frame the relevance of quality research and results within policy context can help to engage policy-makers and make their implications for policy change evident and easily translatable.

Future directions and areas for further study include investigating the influences of research evidence in other areas of pharmacist scope of practice, such as provider status. This work and insights into research evidence engagement can also be compared with pharmacist scope of practice expansion legislation that was introduced but did not become law in other states, to compare the role that it played throughout the scope of practice legislative process.

## Conclusion

By analysing the utilization of research evidence by policy-makers and pharmacy advocates and the barriers researchers face when conceptualizing and advocating for evidence-based scope of practice authority, researchers seeking to influence pharmacist scope of practice and health policy can better understand how to disseminate and implement their work. Utilizing pharmacist prescriptive authority as a proxy for scope of practice policy, this study’s findings emphasize the need to address motivation for research use, capacity for use, engagement, and purpose of research evidence in policy-making. By identifying and creating lasting partnerships with stakeholder groups that can influence policy, as well as addressing common barriers to utilizing research evidence, researchers can adopt dissemination strategies to effectively interact with policy influencers. The recommendations for engaging policy-makers and improving the dissemination of research can be applied not only to broadening pharmacist prescriptive authority, but also to broadening scope of practice as the pharmacist’s role is transformed from traditional dispensing to evolved patient care.

## Supplementary Information

**Additional file 1.** Semi-Structured Interview Guide.

## Data Availability

The datasets used and/or analysed during the current study are available from the corresponding author on reasonable request.

## Abbreviations

COVID-19: Coronavirus disease 2019
NASPA: National Alliance of State Pharmacy Associations
SPIRIT: Supporting Policy In health with Research: an Interventional Trial
ASTHO: Association of State and Territorial Health Officials
SEER: Seeking, Engaging with and Evaluating Research
SAGE: Staff Assessment of enGagement with Evidence
ACOG: American College of Obstetricians and Gynecologists

## References

[1] Dower C, Moore J, Langelier M (2013). Analysis & commentary: it is time to restructure health professions scope-of-practice regulations to remove barriers to care. Health Aff.

[2] Providers C, Reduce C, Disparities GH, Industry P, Reading C, Healthcare H, et al. How Healthcare Reform Is Impacting Primary [Internet]. 2016 [cited 2020 Jul 16]. Available from: https://www.ajmc.com/contributor/sophia-bernazzani/2016/03/how-healthcare-reform-is-impacting-primary-care.

[3] Kirch DG, Petelle K (2017). Addressing the physician shortage: the peril of ignoring demography [Internet]. JAMA J Am Med Assoc.

[4] of American Medical Colleges A. The Complexities of Physician Supply and Demand: Projections From 2018 to 2033. 2020.

[5] Are There Enough Doctors For The Newly Insured? | Kaiser Health News [Internet]. [cited 2020 Jul 16]. Available from: https://khn.org/news/doctor-shortage-primary-care-specialist/.

[6] Dall T, West T. 2017 Update the complexities of physician supply and demand: projections from 2015 to 2030 Final Report Association of American Medical Colleges. 2017.

[7] Gong G, Phillips SG, Hudson C, Curti D, Philips BU (2019). Higher US rural mortality rates linked to socioeconomic status, physician shortages, and lack of health insurance. Health Aff [Internet]..

[8] GUIDANCE TO STATES 1 Lifting Restrictions to Extend the Capacity of the Health Care Workforce during the COVID-19 National Emergency [Internet]. [cited 2020 Jul 16]. Available from: https://www.nga.org/coronavirus/#states.

[9] APhA coronavirus watch: Pharmacists in New York will be ready when the COVID-19 vaccine becomes available | American Pharmacists Association [Internet]. [cited 2020 Jul 16]. Available from: https://www.pharmacist.com/article/apha-coronavirus-watch-pharmacists-new-york-will-be-ready-when-covid-19-vaccine-becomes.

[10] Gradison M (2014). Advocacy: how we can support physician assistants. J Physician Assist Educ.

[11] Brien JMO (2016). How nurse practitioners obtained provider status. Am J Heal Syst Pharm [Internet]..

[12] Carthon JMB, Wiltse Nicely K, Altares Sarik D, Fairman J (2016). Effective strategies for achieving scope of practice reform in Pennsylvania. Policy Polit Nurs Pract [Internet]..

[13] Park J, Han X, Pittman P (2020). Does expanded state scope of practice for nurse practitioners and physician assistants increase primary care utilization in community health centers?. J Am Assoc Nurse Pract [Internet]..

[14] Clement DM (2018). Factors influencing Georgia Legislators’ decision-making on nurse practitioner scope of practice. Policy Polit Nurs Pract [Internet]..

[15] Adams AJ, Martin SJ, Stolpe SF (2011). “Tech-check-tech”: a review of the evidence on its safety and benefits. Am J Heal Pharm [Internet]..

[16] Big scope expansion victories earned in Pennsylvania, Iowa, despite a turbulent year | AOA [Internet]. [cited 2021 May 4]. Available from: https://www.aoa.org/news/advocacy/state-advocacy/pennsylvania-and-iowa-earn-big-victories-to-expand-scope-of-practice?sso=y.

[17] Oliver TR (2006). The politics of public health policy. Annu Rev Public Health [Internet]..

[18] Brownson RC, Chriqui JF, Stamatakis KA (2009). Understanding evidence-based public health policy. Am J Public Health.

[19] Federation of State Medical Boards. Assessing Scope of Practice in Health Care Delivery: Critical Questions in Assuring Public Access and Safety Adopted as policy by the Federation of State Medical Boards in 2005 [Internet]. 2005 [cited 2020 Apr 12]. Available from: http://www.fsmb.org/siteassets/advocacy/policies/assessing-scope-of-practice-in-health-care-delivery.pdf.

[20] Sadeghi-Bazargani H, Tabrizi JS, Azami-Aghdash S (2014). Barriers to evidence-based medicine: a systematic review [Internet]. J Eval Clin Pract.

[21] Haynes B, Haines A (1998). Getting research findings into practice. Barriers and bridges to evidence based clinical practice [Internet]. Br Med J.

[22] Oliver K, Innvar S, Lorenc T, Woodman J, Thomas J (2014). A systematic review of barriers to and facilitators of the use of evidence by policymakers [Internet]. BMC Health Serv Res.

[23] Innvær S, Vist G, Trommald M, Oxman A (2002). Health policy-makers’ perceptions of their use of evidence: a systematic review [Internet]. J Health Serv Res Policy.

[24] Urick BY, Meggs EV (2019). Towards a greater professional standing: evolution of pharmacy practice and education, 1920–2020. Pharmacy [Internet]..

[25] Koehler T, Brown A (2017). Documenting the evolution of the relationship between the pharmacy support workforce and pharmacists to support patient care. Res Soc Adm Pharm.

[26] Avalere Health LLC. Exploring Pharmacists’ Role in a Changing Healthcare Environment. 2014.

[27] Pharmacist Scope of Services [Internet]. [cited 2020 Apr 12]. Available from: https://www.pharmacist.com/sites/default/files/files/APhA—PAPCCScopeofServices.pdf.

[28] Isetts BJ, Schondelmeyer SW, Artz MB, Lenarz LA, Heaton AH, Wadd WB (2008). Clinical and economic outcomes of medication therapy management services: the Minnesota experience. J Am Pharm Assoc.

[29] Mdege ND, Chindove S (2014). Effectiveness of tobacco use cessation interventions delivered by pharmacy personnel: a systematic review. Res Soc Adm Pharm [Internet]..

[30] Nkansah N, Mostovetsky O, Yu C, Chheng T, Beney J, Bond CM (2010). Effect of outpatient pharmacists’ non-dispensing roles on patient outcomes and prescribing patterns. Cochrane Database Syst Rev [Internet]..

[31] Adams ML, Blouin RA. The role of the pharmacist in health care: expanding and evolving. N C Med J. 2017; 78. 10.18043/ncm.78.3.165 28576952

[32] Gardner JS, Miller L, Downing DF, Le S, Blough D, Shotorbani S (2008). Pharmacist prescribing of hormonal contraceptives: results of the Direct Access study. J Am Pharm Assoc.

[33] Lee AJ, Boro MS, Knapp KK, Meier JL, Korman NE (2002). Clinical and economic outcomes of pharmacist recommendations in a Veterans Affairs medical center. Am J Heal Pharm.

[34] Baroy J, Chung D, Frisch R, Apgar D, Slack MK (2016). The impact of pharmacist immunization programs on adult immunization rates: a systematic review and meta-analysis. J Am Pharm Assoc.

[35] Isenor JE, Edwards NT, Alia TA, Slayter KL, MacDougall DM, McNeil SA (2016). Impact of pharmacists as immunizers on vaccination rates: a systematic review and meta-analysis [Internet]. Vaccine.

[36] Tran D, Gatewood S, Moczygemba LR, Stanley DD, Goode JVR (2015). Evaluating health outcomes following a pharmacist-provided comprehensive pretravel health clinic in a supermarket pharmacy. J Am Pharm Assoc.

[37] Beahm NP, Smyth DJ, Tsuyuki RT (2018). Outcomes of urinary tract infection management by pharmacists (RxOUTMAP): a study of pharmacist prescribing and care in patients with uncomplicated urinary tract infections in the community. Can Pharm J [Internet]..

[38] Majercak KR (2019). Advancing pharmacist prescribing privileges: is it time?. J Am Pharm Assoc.

[39] Cranor CW, Bunting BA, Christensen DB (2003). The Asheville project: long-term clinical and economic outcomes of a community pharmacy diabetes care program. J Am Pharm Assoc [Internet]..

[40] Pharmacist Scope of Services [Internet]. [cited 2020 Apr 14]. Available from: https://www.pharmacist.com/sites/default/files/files/APhA—PAPCCScopeofServices.pdf

[41] Giberson S, Yoder S, Lee M. Improving patient and health system outcomes through advanced pharmacy practice. A report to the US surgeon general. [Internet]. Public Health Service. 2011 [cited 2020 Apr 14]. Available from: http://www.uiwpharmacyreview.com/index.php/uiwpr/article/view/29.

[42] Anderson L, Hartung DM, Middleton L, Rodriguez MI (2019). Pharmacist provision of hormonal contraception in the Oregon Medicaid population. Obstet Gynecol [Internet]..

[43] American College of Clinical Pharmacists. State Credentialing Requirements for Expanding Pharmacists’ Scope of Practice. [Internet]. [cited 2020 Apr 12]. Available from: https://www.accp.com/docs/positions/misc/Issue_Brief_State_Credentialing_Requirements.pdf.

[44] American Society of Health System Pharmacists; AHFS Drug Information. Bethesda, MD [Internet]. 2009 [cited 2020 Apr 12];2162. Available from: https://www.ashp.org/New-Practitioner/New-Practitioners-Forum/Resources/Advocacy/Advocacy-Toolkit.

[45] Adams AJ, Weaver KK (2016). The continuum of pharmacist prescriptive authority. Ann Pharmacother [Internet]..

[46] NASPA. Pharmacist Prescribing: Naloxone—NASPA [Internet]. [cited 2020 Apr 12]. Available from: https://naspa.us/resource/naloxone-access-community-pharmacies/.

[47] Pharmacist Prescribing for Tobacco Cessation Medications—NASPA [Internet]. [cited 2019 Feb 20]. Available from: https://naspa.us/resource/tobacco-cessation/.

[48] Pharmacists Provide Access to Care: Contraceptive Prescribing—NASPA [Internet]. [cited 2019 Feb 20]. https://naspa.us/resource/rph-access-contraceptives/.

[49] Adams AJ, Weaver KK (2016). The continuum of pharmacist prescriptive authority. Ann Pharmacother.

[50] Scope of Practice Overview—Scope of Practice Policy [Internet]. [cited 2021 May 4]. Available from: https://scopeofpracticepolicy.org/practitioners-overview/.

[51] Scope of Practice [Internet]. [cited 2021 May 4]. Available from: https://pharmacist.com/Practice/Practice-Resources/Scope-of-Practice.

[52] Redman S, Turner T, Davies H, Williamson A, Haynes A, Brennan S (2015). The SPIRIT action framework: a structured approach to selecting and testing strategies to increase the use of research in policy. Soc Sci Med.

[53] Williamson A, Redman S, Haynes A, Barker D, Jorm L, Green S (2014). Supporting policy in health with research: an intervention Trial (SPIRIT)—protocol for a stepped wedge trial. BMJ Open.

[54] Cardno C (2019). Policy document analysis: a practical educational leadership tool and a qualitative research method. Educ Adm Theory Pract.

[55] NASPA. Pharmacist Prescribing: Tobacco Cessation Aids—NASPA [Internet]. [cited 2020 Apr 12]. Available from: https://naspa.us/resource/tobacco-cessation/.

[56] Naloxone Access in Community Pharmacies—NASPA [Internet]. [cited 2019 Feb 20]. Available from: https://naspa.us/resource/naloxone-access-community-pharmacies/.

[57] Pharmacists Authorized to Prescribe Tobacco Cessation Therapy in More States—NASPA [Internet]. [cited 2020 Apr 12]. Available from: https://naspa.us/2017/06/pharmacists-authorized-to-prescribe-tobacco-cessation-therapy-in-more-states/.

[58] Tobacco Control Network. Access to Tobacco Cessation Medication through Pharmacists [Internet]. 2017 [cited 2020 Apr 12]. Available from: https://www.astho.org/Prevention/Tobacco/Tobacco-Cessation-Via-Pharmacists/.

[59] State Public Health | ASTHO [Internet]. [cited 2020 Apr 12]. Available from: https://www.astho.org/StatePublicHealth/States-Authorize-Pharmacists-to-Prescribe-Dispense-Contraceptives/06-06-19/.

[60] Zeeck E. State Public Health | ASTHO [Internet]. Association of State and Territorial Health Officials. 2017 [cited 2020 Apr 12]. Available from: https://www.astho.org/StatePublicHealth/States-Authorize-Pharmacists-to-Prescribe-Dispense-Contraceptives/06-06-19/.

[61] Adams AJ, Hudmon KS (2018). Pharmacist prescriptive authority for smoking cessation medications in the United States. J Am Pharm Assoc.

[62] NASPA. Pharmacists Authorized to Prescribe Birth Control in More States—NASPA [Internet]. News. 2017 [cited 2020 Apr 12]. Available from: https://naspa.us/2017/06/pharmacists-authorized-to-prescribe-tobacco-cessation-therapy-in-more-states/.

[63] Bureau UC. 2010 Census Regions and Divisions of the United States [Internet]. 2010 [cited 2021 May 4]. Available from: https://www.census.gov/geographies/reference-maps/2010/geo/2010-census-regions-and-divisions-of-the-united-states.html.

[64] Makkar SR, Brennan S, Turner T, Williamson A, Redman S, Green S (2016). The development of SAGE: a tool to evaluate how policymakers’ engage with and use research in health policymaking. Res Eval.

[65] Brennan SE, McKenzie JE, Turner T, Redman S, Makkar S, Williamson A (2017). Development and validation of SEER (Seeking, Engaging with and Evaluating Research): a measure of policymakers’ capacity to engage with and use research. Heal Res Policy Syst [Internet]..

[66] Zoom Video Conferencing [Internet]. [cited 2020 Apr 15]. Available from: https://software.sites.unc.edu/zoom/.

[67] Bowen GA (2008). Naturalistic inquiry and the saturation concept: a research note. Qual Res [Internet]..

[68] Hsieh HF, Shannon SE (2005). Three approaches to qualitative content analysis. Qual Health Res.

[69] DeCuir-Gunby JT, Marshall PL, McCulloch AW (2011). Developing and using a codebook for the analysis of interview data: an example from a professional development research project. Field Methods [Internet]..

[70] Decuir-Gunby JT, Marshall PL, Mcculloch AW. Developing and using a codebook for the analysis of interview data: an example from a professional development research project. [cited 2020 May 1]; Available from: http://fm.sagepub.com.

[71] Livet M, Yannayon M, Sheppard K, Kocher K, Upright J, McMillen J (2017). Exploring provider use of a digital implementation support system for school mental health: a pilot study. Adm Policy Ment Heal Ment Heal Serv Res.

[72] O’Brien BC, Harris IB, Beckman TJ, Reed DA, Cook DA (2014). Standards for reporting qualitative research: a synthesis of recommendations. Acad Med.

[73] O’Sullivan TA, Jefferson CG. A review of strategies for enhancing clarity and reader accessibility of qualitative research results. Am J Pharm Educ. 2020;84(1). 10.5688/ajpe7124 PMC7055402 32292189

[74] Birt L, Scott S, Cavers D, Campbell C, Walter F (2016). Member checking: a tool to enhance trustworthiness or merely a nod to validation?. Qual Health Res [Internet]..

[75] Considerations L. Changes in Healthcare Professions’ Scope of Practice. 2006; Available from: https://www.ncsbn.org/ScopeofPractice_09.pdf.

[76] Lebuhn R. Reforming scopes of practice. Citiz Advocacy Cent [Internet]. 2010. Available from: cac@cacenter.org.

[77] Fairman JA, Rowe JW, Hassmiller S, Shalala DE (2011). Broadening the scope of nursing practice [Internet]. New Engl J Med Massachussetts Med Soc.

[78] Martens PJ, Roos NP (2005). When health services researchers and policy makers interact: tales from the tectonic plates. Healthc Policy [Internet]..

[79] Choi BCK, Pang T, Lin V, Puska P, Sherman G, Goddard M (2005). Can scientists and policy makers work together? [Internet]. J Epidemiol Commun Health..

[80] Brownson RC, Royer C, Ewing R, McBride TD (2006). Researchers and policymakers: travelers in parallel universes. Am J Prevent Med..

[81] Breland JY, Quintiliani LM, Schneider KL, May CN, Pagoto S (2017). Social media as a tool to increase the impact of public health research. Am J Public Health [Internet]..

[82] Allen HG, Stanton TR, Di Pietro F, Moseley GL (2013). Social media release increases dissemination of original articles in the clinical pain sciences. PLoS One [Internet]..

[83] Dong JK, Saunders C, Wachira BW, Thoma B, Chan TM. Social media and the modern scientist: a research primer on social media-based research, dissemination, and sharing. Afr J Emerg Med. 2020; 10.1016/j.afjem.2020.04.005 PMC7718451 33304794

