# Supplementary material for: Use of research evidence varied in efforts to expand specific pharmacist autonomous prescriptive authority: an evaluation and recommendations to increase research utilization
Source: Health Res Policy Syst. 2022 Jan 3;20:1. doi: 10.1186/s12961-021-00789-9 (PMC8721476; doi:10.1186/s12961-021-00789-9)
Supplement: Supplementary file 1 — Additional file 1. Semi-Structured Interview Guide. [file 12961_2021_789_MOESM1_ESM.pdf]

**Interview Guide: Use of Evidence in Prescriptive Authority**

GREETING AND CONSENT:

My name is XX and I am a YY with UNC Eshelman School of Pharmacy. Is now still a good time to talk?

Before I begin, I wanted to share more information with you to ensure you are informed and consent to the interview.

- The purpose of this interview is to understand how you did or did not use evidence to create this policy.
- We wanted to speak with you about your role in the development and passage of **name of policy** in **state*.*
- This interview should take 30-60 minutes to complete.
- Your participation is completely voluntary.
- We will ask some planned questions and ask follow-up questions based on your answers. You may skip any questions or stop taking part in the interview at any time.
- We would like to record the conversation to ensure that your thoughts are accurately captured.
- The recording will only be accessed by select study team members and will be erased as soon as we are able to review it and make sure that our notes are complete.
- We will not share information that is specifically connected to you in any reports or findings. All information collected will be summarize across all the interviews for reporting.

Do you consent to participation in this study?

Do you consent to being recorded?

Do you have any questions or concerns before we start?

We appreciate you agreeing to help us understand how research evidence can be better utilized in the policy-making process. For the purposes of this study, we are defining research as the analyses of data or concepts found in peer reviewed papers, technical monographs or books, or in grey literature such as internal studies and evaluations, and reports on authoritative websites. Advice from researchers is considered to be *research-informed information*, but not research per se. We will use the terms *research*, *research findings* and *research evidence* interchangeably.

Additionally, when we ask about the "policy", we are referring to the statewide protocol or legislation regarding **specific state's* *category** prescriptive authority/**name of the bill/law*.*

BACKGROUND:

1. Can you tell me about the **category** prescriptive authority we will discuss today?

   <u>Prompts:</u>

   - **could ask clarify questions that came from literature search**

2. What was your role in the development of this policy? This can include anything from conceptualization to passage of this policy.
3. What types of research were found when considering/developing this policy?

CATALYST:

4. What prompted the use of research in the development of this policy?

   <u>Prompts</u>

   - What was the policy or program need for research? (need for further education, supporting the case for the policy, research guided political agenda, or something else?)
   - Was there new research that came out that was relevant to the policy?

USE/OUTCOME:

5. Tell me how you or others used research to inform the development of this policy.

   <u>Prompts:</u>

   - Give time to unpack all the ways in which research contributed to the development of the policy.
   - Can you describe how you used research in that way?

6. I'm going to talk about four different ways that research can be used and after each one I'd like you to tell me if you used research in that way or not.
   a. How was research used to help you understand how to think about the issue of **category**?
      i. Can you describe how you used research in that way?
   b. How was research used to make decisions about the policy's content or direction?
      i. Can you describe how you used research in that way?
   c. How was research used to persuade others to support this policy? This can include anything from ideological to tactical support.
      i. Can you describe how you used research in that way?
   d. How was research used because your organization required you to use research?
      i. Can you describe how you used research in that way?

e. How was research used in other ways?

CAPACITY:

7. Did you work with an organization/institution for the development or implementation of this policy?
8. If so, which organization/institution, and how did your organization or institution facilitate the use of research for this policy?

   Prompts

   - Are there protocols in place to use research?
   - Do you have leaders that support or model the use of research?
   - What resources were in place to access and interact with research?
9. What do you feel could have been done to enhance your capacity (knowledge or skills) to use research in relation to this policy?

ENGAGEMENT ACTIONS:

10. How did you access research for this policy?

    Prompts

    - How did you or others look for research to inform this policy?
    - What types of research were found when looking for this policy? (population trends, databases, peer-reviewed publications, gray literature)
11. How relevant was available research to the topic of this policy?

    Prompts:

    - How did you, or others work out whether the research was relevant or not?
    - What was the quality of the research that was found? (population, study design, stats/analysis)
12. How did you interact with researchers when developing this policy?
13. Did you, or others, consider conducting or commissioning more research to support the development or passage of this policy?

    Prompts

    - If so, did you partner with/commission researchers, organize a committee working group, or internally analyze research and data regarding the policy?
    - What was the reasoning behind considering conducting/commissioning more research?

BARRIERS

14. What were barriers to using research in the consideration, development, and/or passage of this policy?

    Prompts:

- What were some things that made it difficult to use research?
- From the perspective of the individual, organizational, agency, political, topical, temporal
- If barriers have been identified, determine cause

15. What impact did those barriers have on this prescriptive authority policy?

    Prompts:

    - Were those barriers surmountable, or did they completely prevent the use of research?

CONCLUSION:

16. What are your recommendations for researchers looking to make evidence to influence policy?
17. What was hoped to be gained from the research used to craft this policy? What was the motivation for using research in relation to this prescriptive policy?

That concludes the main part of the interview!

18. What other comments about your experience with research and *this policy* would be helpful for us to know?

Do you have about this study or anything we talked about today? If not, thank you so much for your time today in speaking with us! Your perspective and contributions will help us better understand how researchers can best support the endeavors of policy makers and pharmacy advocates in improving patient care and utilizing pharmacists to their full potential. Here is my contact information if anything arises **provide contact info**, have a good day!
